# Protective Effects of *Descurainia sophia* against Gentamicin Induced Nephrotoxicity in Rats

**DOI:** 10.22037/ijpr.2020.112105.13535

**Published:** 2021

**Authors:** Hassan Askari, Noora Enayati, Mohammad Mehdi Ahmadian-Attari, Mahmood Bakhtiyari, Amirhesam Alirezaei

**Affiliations:** a *Gastroenterohepatology Research Center, Shiraz University of Medical Sciences, Shiraz, Iran. *; b *Department of Photochemistry, Medicinal Plants and Drug Research Institute, Shahid Beheshti University, Tehran, Iran. *; c *Evidence-based Phytotherapy and Complementary Medicine Research Center, Alborz University of Medical Sciences, Karaj, Iran. *; d *Non-communicable Diseases Research Center, Alborz University of Medical Sciences, Karaj, Iran. *; e *Department of Community Medicine, School of Medicine, Alborz University of Medical Sciences, Karaj, Iran. *; f *Clinical Research and Development Center, Shahid Modarres Hospital, Department of Nephrology, Shahid Beheshti University of Medical Sciences, Tehran, Iran.*

**Keywords:** Renal toxicity, Nephrotoxicity, Descurainia Sophia, Gentamicin, Acute kidney injury

## Abstract

Several studies have tried to find an efficient agent to prevent or reverse gentamicin (Gm) induced acute kidney injury (AKI). In this study, we assessed the potential renal protective effects of *Descurainia sophia *(L.) Webb ex Prantl against Gm-induced nephrotoxicity in rats. Thirty-five male Wistar rats were categorized in five groups (n = 7 per group). Control group was treated with normal saline. In four experimental groups, the rats were initially treated with normal saline (A), 800 (B), 1600 (C) and 2400 (D) mg/kg *Descurainia sophia respectively for 28 days. *After that, the rats of experimental groups were treated with Gm (80 mg/Kg) for 7 consecutive days. Blood and urine markers, as well as apoptosis and histological features were determined. Serum BUN, creatinine, cholesterol, and triglycerides level, as well as urinary excretion of Na^+^ significantly increased in group A. Furthermore, Gm induced inflammatory cells infiltration, apoptosis, and renal cells injuries in rats were pretreated with normal saline (group A). However, in the rats pretreated with *Descurainia sophia* extract (groups B, C, and D, there were significant and dose-dependent reductions in serum BUN, creatinine, cholesterol and triglyceride, urinary Na^+^ excretion, apoptosis rate, and inflammatory cells infiltration in renal tissues. Overall, *Descurainia sophia* showed significant protective effects against Gm-induced AKI by alleviating biochemical and histological markers of renal toxicity.

## Introduction

Drug-induced acute kidney injury (AKI) is a dreadful condition comprising around 60% of all renal toxicities (1). On the other hand, 3.2% of all adverse reactions induced by drugs have been against renal cells (2). Intravenous antibiotics including vancomycin and gentamicin (Gm) have been responsible for about 29% of all drug-induced AKIs (2, 3). Gm is a widely used antibiotic belonging to aminoglycosides used to treat infections caused by gram-negative bacteria (4). Despite its high anti-bacterial efficiency, Gm is deterred due to potential renal toxicity (5). AKI has been noted in as high as 10-25% of the patients treated with Gm; even those received a single dose of this drug (6-9). Renal tubules uptake around 10% of intravenously administrated Gm which is considered to be the primary source of renal structural and functional alternations (5).

It is believed that oxidative stress plays a major role in Gm-induced AKI; nevertheless, a multifactorial phenomenon seems to be more plausible. The antioxidant powers in the kidneys exposed to Gm are reduced leading to renal tubular and epithelial cells necrosis and apoptosis. Gm also promotes cellular death by activating mitochondrial initiated apoptotic pathway and B cell lymphoma-2 (Bcl-2) associated protein X (Bax) (10). Furthermore, renal tubular injury in Gm-associated renal dysfunction is associated with attenuated glomerular filtration rate (8). 

Extensive efforts have been dedicated to find agents that prevent or at least reduce the nephrotoxicity effects of Gm. In this regard, a recent study has reviewed 54 nephroprotectants evaluated in animal models (11). These agents can be categorized in various subclasses including anti-oxidative and anti-inflammatory agents, diuretics, calcium channel blockers, and inhibitors of aminoglycoside tubular uptake (11). In particular, plants extracts with anti-oxidant and anti-inflammatory properties have been always under attention as nephroprotective agents (12). 

*Descurainia sophia *is a plant member of Brassicaceae family. This plant, which is also known as “flixweed” or “Khak-e-sheer” (in Iranian traditional medicine), grows in a wide geographic belt expanding throughout Asia, North Africa, North America, and Europe (13). This plant has been used to alleviate asthma, cough, cardiac dysfunction, and edema in Iranian, Chinese and Indian traditional medicines (13-15). It has been shown that at least 26 biologically active elements regulating metabolism of amino acids, metabolic pathways, and energy expenditure are present in the aqueous seed extract of * Descurainia sophia*  (16). In another study, Lee et al isolated 14 active components from the seeds of *Descurainia sophia* and investigated their anti-proliferative and anti-inflammatory effects (17). Alcoholic extract of * Descurainia sophia*  has been shown to present antipyretic, anti-inflammatory, analgesic (18), anti-tumor, anti-proliferative (19, 20) and anti-asthma (21) effects. In clinical terms, the beneficial role of * Descurainia sophia*  has been reported in chronic heart diseases (22) and functional constipation (23) as well. 

A variety of agents with wide-range biological activities such as dapagliflozin (5), sika deer antler protein (8), *costus afer*, hesperidin, grape seed extract, pinocembrin, Zn, *Portulaca oleracea* extract, dipyridamole, α-tocopherol (vitamin E), troxerutin, cobalamin, Rudgea viburnoides and apocynin have been evaluated as nephroprotectants in different models of AKI (24-37). All of these agents have resulted in variable results and side effects. However, despite diverse anti-oxidative and anti-inflammatory effects, the protective role of *Descurainia sophia* against AKI induced by Gm has not been studied. Therefore, we aimed to use an animal model of Gm-induced AKI to assess if extract of this plant can prevent or at least mitigate renal adverse effects induced by this antibiotic. 

## Experimental

To assess the protective effects of *Descurainia sophia *on Gm-induced renal insufficiency, 35 male Wistar rats were treated with the antibiotic for 7 consecutive days. The rats were provided by Tehran University of Medical Sciences animal house. The rats’ weight ranged as 200-250 grams, and they had free access to fresh water and food. They were kept in standard cages at room temperature (25 ± 3 °C), 50-60% moisture and under natural 12-hour dark/light cycle. 

Also, based on old Persian pharmaceutical manuscripts (24),* Descurainia sophia* has been reported as a general antidote to neutralize toxicity of toxic herbs. Moreover, previous studies have shown that this herb has hepatoprotective activity against hepatotoxicity induced by acetaminophen (25) Therefore, and as a next step, our study has focused on the nephroprotective activity of this herb.

*Plant extraction and standardization*

*Descurainia sophia* seed was prepared from Tehran botanical market and authenticated by the Herbarium of School of Traditional Medicine, Shahid Beheshti University of Medical Sciences (HMS-520). hydroalcoholic extract of *Descurainia sophia* was prepared using a previously described method (26). Briefly, the seeds were ground and extracted by macerating the powder in 96% ethanol (1:1) for 24 h. Then, the extract was concentrated using rotary evaporation system (Hiedolph, Geremany) at 60°C. The extract was then left to dry in a desiccator. Based on a previously described method (27) and to standardize the extract, total phenolic content was determined via spectrophotometry and reported as gallic acid equivalent.

The extract consisted of an upper oil phase and a lower solid phase. The yield of extraction was 8.57% (6.2% for solid phase). The result, which is mean of three replicates showed that total phenolic compounds of the extract is 95 mg/g. 

*Experimental groups*

**Figure 1:**
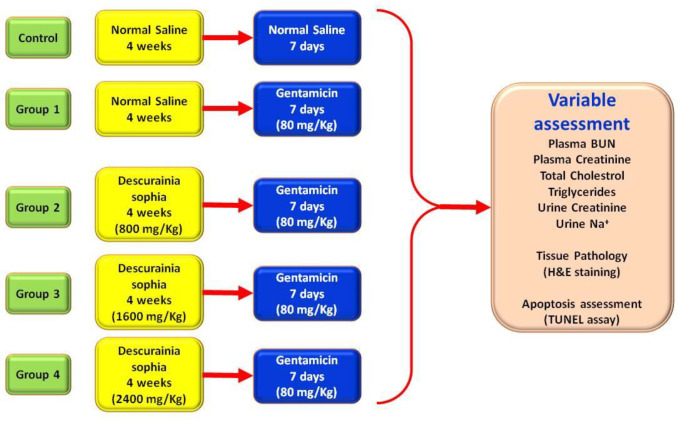
Study protocol and treatment groups. Wistar rats were categorized into 5 separate groups: control group was treated with normal saline for 4 weeks and then 7 days, Group 1 was treated by normal saline for 4 weeks followed by 7 days of 80 mg/Kg gentamicin, Groups 2, 3, and 4 were treated with 800, 1600, and 2400 mg/Kg *Descurainia sophia *respectively for 4 weeks followed by 7 consecutive days of treatment with 80 mg/Kg Gentamicin

The experimental groups included 1) control: administrating normal saline throughout the study; 2) group 1: four weeks normal saline, 3) group 2: four weeks of 800 mg/Kg * Descurainia sophia *extract*, *4) Group 3: four weeks of 1600 mg/Kg of * Descurainia sophia *extract, and 5) Group 4: four weeks of 2400 mg/Kg * Descurainia sophia *extract. At the end of four weeks, the rats in all the experimental groups (i.e. group 1 to 4) received Gm injections (80 mg/Kg) for 7 consecutive days (Figure 1).

*Sample collection and apoptosis evaluation*

The rats were sacrificed at the end of treatments. The kidneys were removed, fixed in 4% formaldehyde solution and stored in 70% ethanol solution until the day of tissue processing and apoptosis evaluation. We used TUNEL apoptosis detection kit (Abbkine Scientific Co,USA) based on the manufacturer’s instructions. In this assay, DNA nicks and fragments are detected using florescence microscopy. The results were recorded as the percentage of the damaged cells. 

*Urine and blood biochemistry analysis*

Biochemical assays were performed using standard protocols in a clinical laboratory. Plasma BUN, creatinine, cholesterol, triglyceride and urine creatinine levels were measured using spectrophotometric methods (Pars Azmon, Iran) on a calibrated automatic biochemistry analyzer (ELITech Group Clinical Systems, France). Blood and urine sodium concentrations were determined using a closed electrolyte analyzer (Caretium, China). Measurements were performed as baseline (i.e. before plant extract administration, day 1), at the day 28^th^ (i.e. before starting Gm), and at the day 35^th^ (i.e. the end of study).

*Statistical analysis*

Comparison between the measurement steps was done using repeated measure analysis of variances (ANOVA). The confidence interval level was considered 95%. TUNEL test results were compared between the groups using ANOVA and HSD-Tukey test. 

## Results

*Effects of Descurainia sophia on BUN and creatinine level*

**Figure 2:**
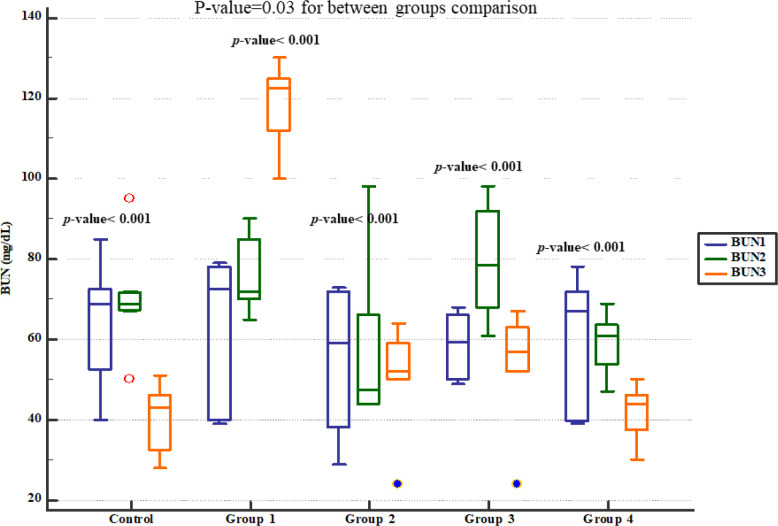
Blood urea nitrogen plasma levels in study groups. BUN1, 2 and 3 represent serum levels at baseline (day 1), the end of *Descurainia sophia* extract treatment (day 28^th^), and the end of study (i.e. after 7 days of gentamicin treatment, day 35^th^) respectively. There was a significant change in serum BUN levels at different steps (*p*-value < 0.001). Further between groups comparison showed that there was a significant difference between groups treated in different manners (*p*-value= 0.030)

**Figure 3:**
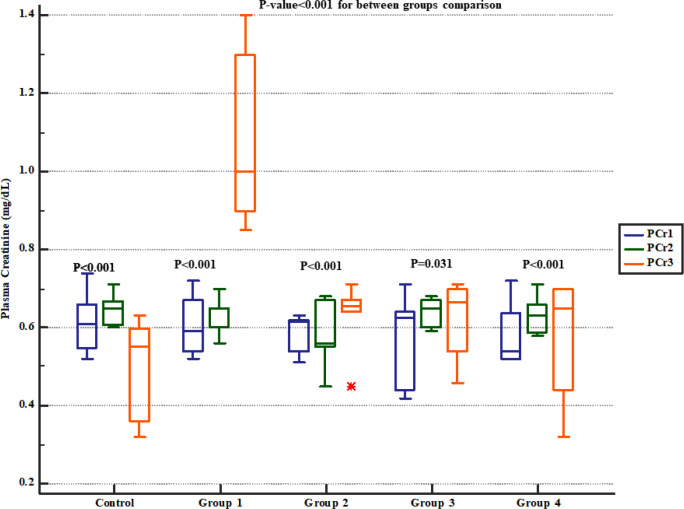
Serum creatinine levels in study groups. PCr1, 2 and 3 represent serum levels at baseline (day 1), the end of *Descurainia sophia* extract treatment (day 28^th^), and the end of study (i.e. after 7 days of gentamicin treatment, day 35^th^) respectively. There was a significant change in plasma creatinine levels between steps of the study (*p*-value< 0.001). Further between groups comparison showed that there was a significant difference between groups treated in different manners (*p*-value< 0.001).

Serum BUN level significantly increased in group 1 (four weeks normal saline and then treated 7 days Gm). However, pre-treatment with either 800, 1600 or 2400 mg/kg of * Descurainia sophia *dose-dependently prevented elevation of BUN following Gm treatment in Wistar rats with the most efficient dose that was 2400 mg/Kg (Figure 2). The rats in group 1 also had significantly increased serum creatinine level while those received various doses of * Descurainia sophia *showed significantly lower creatinine level with the lowest level observed in 2400 mg/kg group (Figure 3). 

*Effects of Descurainia sophia on urinary Na* ^+^ *concentration and fractional excretion of sodium (FeNa* ^+^ *)*

**Figure 4:**
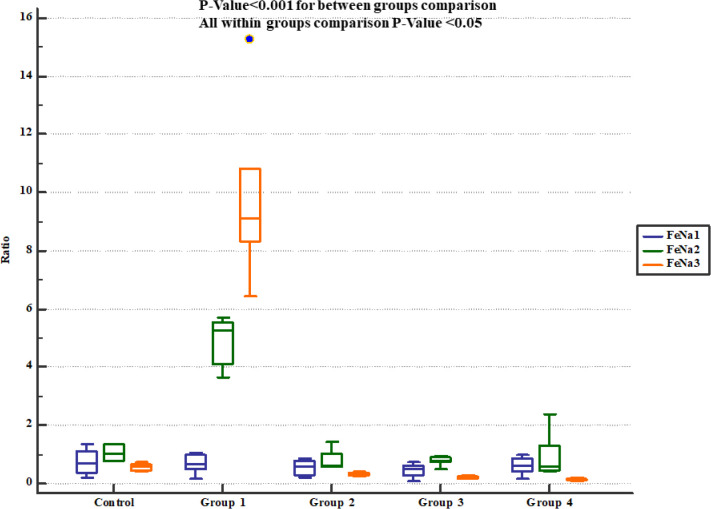
Urine sodium (Na^+^) concentration in study groups. UNa1, 2 and 3 represent serum levels at baseline (day 1), the end of *Descurainia sophia* extract treatment (day 28^th^), and the end of study (i.e. after 7 days of gentamicin treatment, day 35^th^) respectively. There was a significant change in urine sodium concentrations comparing steps of the study (*p*-value< 0.001). Further within group comparison showed that there was a significant difference between groups treated in different manners (*p*-value< 0.001).

**Figure 5:**
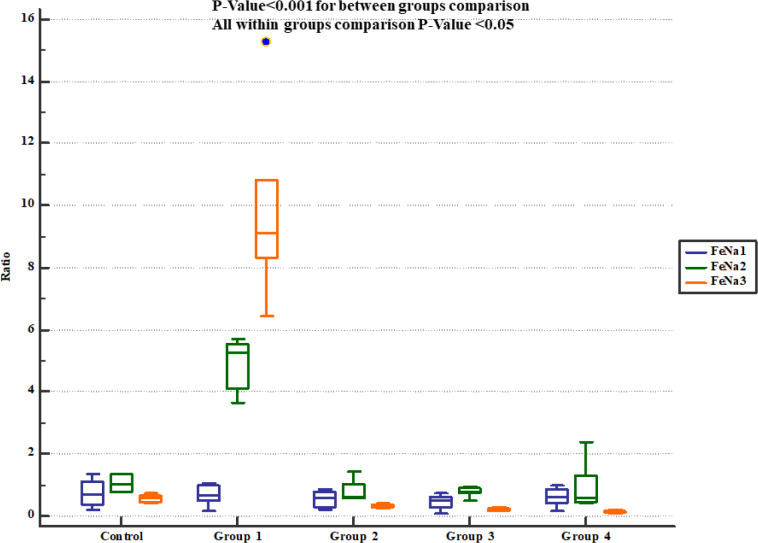
Fractional sodium excretion (FeNa^+^) in study groups. FeNa1, 2 and 3 represent serum levels at baseline (day 1), the end of *Descurainia sophia* extract treatment (day 28^th^), and the end of study (i.e. after 7 days of gentamicin treatment, day 35^th^) respectively. There was a significant change in FeNa^+^ comparing steps of the study (*p*-value< 0.001). Further between groups comparison showed that there was a significant difference between groups treated in different manners (*p*-value< 0.001)

The rats received normal saline showing significant increase in urinary Na^+^ concentration and fractional excretion of sodium (FeNa^+^). On the other hand, pre-treatment with either 800, 1600 or 2400 mg/Kg of * Descurainia sophia *significantly decreased urine Na^+ ^concentration (Figure 4) and FeNa^+^ (Figure 5) at all the studied doses. 

*Effects of Descurainia sophia on lipid profile*

**Figure 6:**
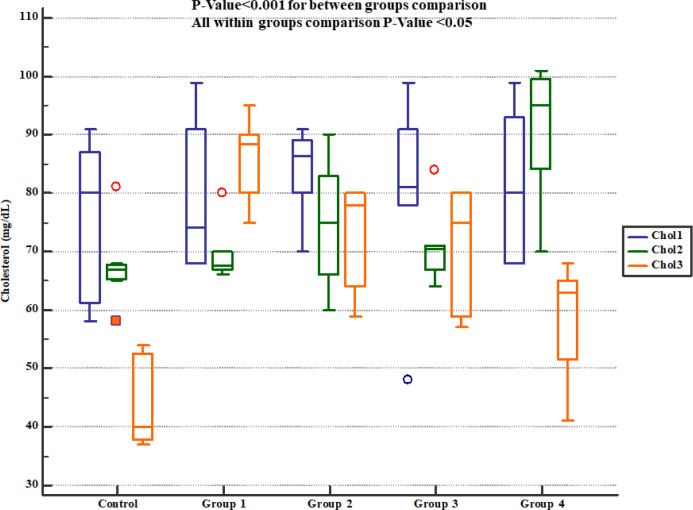
Serum cholesterol levels in study groups. Chol1, 2 and 3 represent serum levels at baseline (day 1), the end of *Descurainia sophia* extract treatment (day 28^th^), and the end of study (i.e. after 7 days of gentamicin treatment, day 35^th^) respectively. There was a significant change in plasma cholesterol levels comparing steps of the study (*p*-value= 0.001). Further between group comparison showed that there was a significant difference between groups treated in different manners (*p*-value< 0.001).

**Figure 7:**
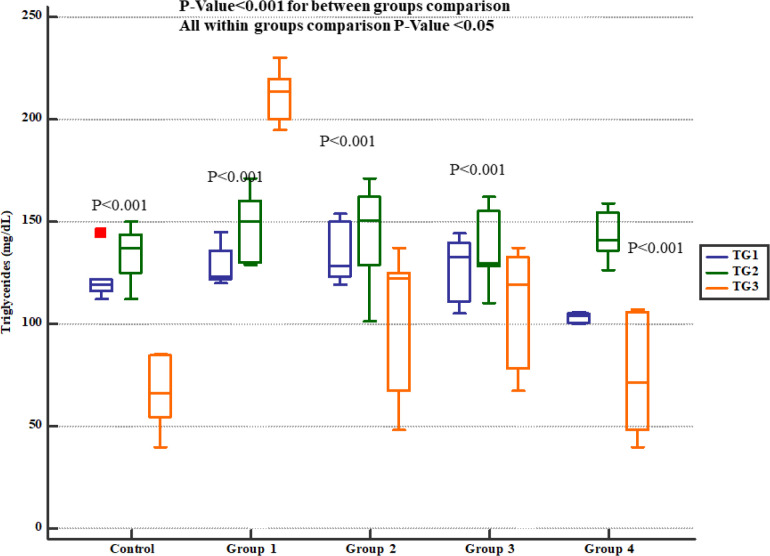
Serum triglycerides levels in study groups. TG1, 2 and 3 represent serum levels at baseline (day 1), the end of *Descurainia sophia* extract treatment (day 28^th^), and the end of study (i.e. after 7 days of gentamicin treatment, day 35^th^) respectively. There was a significant change in plasma triglycerides level comparing different steps of the study (*p*-value< 0.001). Further within group comparison showed that there was a significant difference between groups treated in different manners (*p*-value< 0.001).

The serum levels of cholesterol (Figure 6) and triglycerides (Figure 7) levels significantly increased at the day 35^th^ (i.e. 7 days after Gm exposure) but Pre-treatment with either 800, 1600 or 2400 mg/kg * Descurainia sophia *prevented increments in serum cholesterol and triglycerides levels in Wistar rats. All the doses were significantly efficient; however, the most potent effects were noticed in the dose of 2400 mg/Kg. 

*Effects of Descurainia sophia on apoptosis of kidney cells*

**Figure 8:**
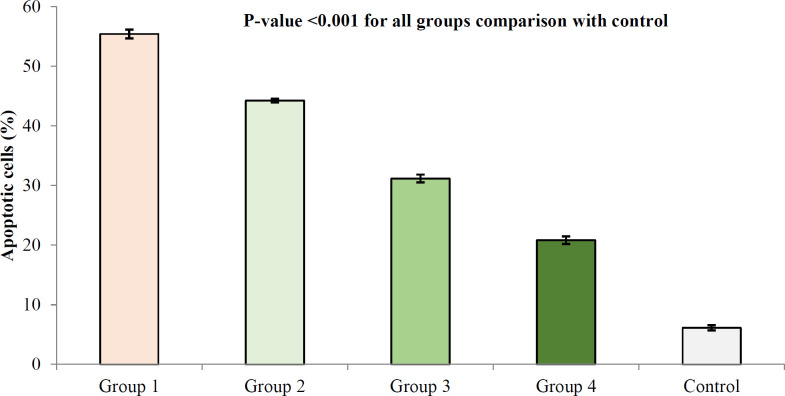
TUNEL apoptosis assay in kidney tissue in different study groups. At the end of study, the animals were sacrificed and kidney tissues were evaluated for apoptosis using TUNEL assay. The study groups included control, and four experimental groups. Group 1 received normal saline + gentamycin treatment. The three other experimental groups received *Descurainia sophia* extract (Group 2: 800 mg/Kg; Group 3: 1600 mg/Kg; and Group 4: 2400 mg/Kg). Analysis of variances showed that there was a significant difference in the percentage of apoptotic cells between the groups (*p*-value< 0.001); HSD-Tukey test, for separate comparisons, also showed significant differences between groups (*p*-value< 0.001 for each double comparisons). *** If *P*-value <0.001 for each comparison with control group

Gm. treatment resulted in the apoptosis of kidney cells; however, treatment with *Descurainia sophia *pre-administration dose-dependently protected renal cells from Gm induced apoptosis (Figure 8). 

## Discussion

 However, there were significant and dose-dependent reductions in serum BUN, creatinine, cholesterol and triglyceride, urinary Na^+^ excretion, apoptosis rate, and inflammatory cells infiltration in renal tissues. Other studies have also evaluated protective effects of various agents against Gm-induced AKI and reported similar findings as increased serum BUN and creatinine levels in animal models (5, 8, 32). Increased levels of BUN and creatinine in the context of Gm-induced renal impairment have been correlated with prominent aberrations in renal function and structure (33). In line, Gm administration for 8 days reduced creatinine clearance and renal blood flow in previous reports (32, 34). The rats exposed to Gm have also shown dose-dependent decline in glomerular filtration rate (29, 35). In support, Troxerutin,a plant flavonoid, was able to correct alternations in urinary protein and albumin levels, as well as serum creatinine and BUN in part by augmenting glomerular filtration rate (29). Although elevations in serum BUN and creatinine indicate renal functional impairment, these traditional markers may not be adequately sensitive to reveal renal toxicity in early phases (36). Some novel markers including urinary neutrophil gelatinase associated protein (NGAL), β_2_-microglobulin, albumin, clusterin, glucose excretion, histological findings, urinary level of kidney injury molecule-1 (KIM-1) and alpha glutathione S-transferase have been suggested to develop a multisystem model for assessing drug induced renal injury (36-38). In mice and HEK293 cells, Gm exposure increased urinary levels of KIM-1, NGAL, and Cys-C activities (8, 29). In addition, as early as four days post Gm-exposition, increased expression of four miRNAs (miR-138-5p miR-1971, miR-218-1-3p, and miR-489) predicted Gm-induced toxicity before elevation of serum BUN and creatinine (39). With advancements in diagnostic methods, novel biomarkers have been proposed to be used as alternatives to serum BUN and creatinine to early detect drug-induced AKI. In current study, pre-treatment with either 800, 1600 or 2400 mg/Kg of * Descurainia sophia *significantly decreased urine Na^+ ^concentration and FeNa^+^ at all the studied doses. In accordance, Gm treatment exerted similar patterns in Na^+ ^levels in previous studies (34, 40). Also previous experiments showed that Gm treatment significantly increased FeNa^+^ level (41, 42). Alternations in electrolytes distribution in Gm-induced AKI can be related to the depressed activity of Na^+^, K^+^-ATPase pump in proximal renal tubules correlating with higher Na^+^ excretion (43). Nevertheless, Sugarman et al described that Gm had no significant impact on the activity of this transporter in renal medullary or cortical cells (35). Gm-induced accumulation and release of Na^+^ and K^+^ respectively from renal proximal tubular cells can also contribute to the disturbed electrolyte turnover in Gm-induced nephrotoxicity (44). In fact, the concentration dysfunction in tubular cells, rather than defects in sodium excretion disturbance, has been suggested as the main cause of disturbed Na^+^ distribution induced by Gm (35). According to studies, Gm damages tubular tissue in kidneys resulting in functional disturbances of tubular cells in regulating the balance of electrolytes and other molecules (45). Trace elements perform important biological roles in the body including participation as structural elements in enzymes, proteins, and hormones (46), and using agents protecting electrolyte disturbance can result in healthy renal and other organs functions. Pre-treatment with either 800, 1600, or 2400 mg/kg *Descurainia sophia *prevented increments in serum cholesterol and triglycerides levels in Wistar rats. All the doses were significantly efficient; however, the most potent effects were noticed in the dose of 2400 mg/Kg. In line with this, Anandan et al in their study also noted that hesperidin effectively prevented Gm-induced elevations of serum cholesterol, free fatty acids, and triglycerides in rats (30). It has been shown that Gm can damage lysosomes resulting in high levels of membrane lipids (sphingomyelin and sphingosine) in serum (47). However, studies evaluating the effects of Gm on lipid profile and its interaction with renoprotective pathways are scare and more studies are needed in this area. Moreover, the results of this study showed that treatment with *Descurainia sophia *pre-administration dose-dependently protected renal cells from Gm induced apoptosis. This observation was in line with a previous report indicating higher apoptosis rate in Gm-induced renal cells (29). Accordingly, after 14 days of administration, Gm induced apoptosis in renal tubular cells in rats (5). In addition to *in-vivo* models, Gm also induced dose-dependent apoptosis in HEK293 (1, 8) and HK-2 (1) kidney cell lines. Gm was shown to activate Bax; a proapoptotic member of Bcl-2 family, which subsequently triggers caspase-3 dependent cellular death (48). Increased expression of Fas ligand (CD95) in renal cells can also contribute to Gm-induced apoptosis (32). In accordance with our results, * Descurainia sophia *extract reduced apoptosis rate in cardiomyocytes of rat model of cardiac disease which was concomitant with depressed levels of Bax and caspase 3 (22). In A549 tumor cells, *Descurainia sophia* seed extract induced the expression of death receptors 4 (DR4) and 5 (DR5) through activating endoplasmic reticulum stress response pathway (49). In another study, dapagliflozin inhibited Gm-induced renal tubular cells apoptosis in part by increasing miR-21 and decreasing miR-181a expressions (5). Overall, the exact anti-apoptotic mechanisms of *Descurainia sophia *in Gm-induced renal injury are to be elucidated.

**Figure 9:**
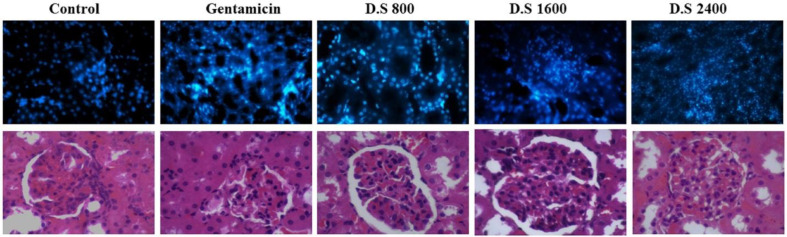
H and E tissue staining and scoring of kidney tissues. At the end of study, the animals were sacrificed and kidney tissues were evaluated for the percent of damaged cells. The study groups included control, and four experimental groups. Group 1 received normal saline + gentamycin treatment. The three other experimental groups received* Descurainia sophia* extract (Group 2: 800 mg/Kg; Group 3: 1600 mg/Kg; and Group 4: 2400 mg/Kg). Analysis of variances showed that there was a significant difference in the percentage of damaged cells between the groups (p-value< 0.000). HSD-Tukey test, for separate comparisons, also showed significant differences between groups (*p*-value< 0.05 for each double comparisons).

Finally, Gm treatment for 7 consecutive days resulted in renal cellular damage and infiltration of inflammatory cells in kidney tissue in the rats receiving saline. Pre-treatment with *Descurainia sophia *significantly reduced cellular injuries and recruitment of inflammatory cells to kidney tissue (Figure 9). An interstitial nephritis may also accompany the tubular damage by Gm (53-57). A lymphoproliferative phenomenon was also observed in renal tissue of Gm-exposed rats in a study by Oliveira et al (50). In other studies, histological characteristics have been noted in Gm-treated kidneys as degenerative changes (32, 51) and vaculation (5) in tubular epithelial cells (5, 36) and glomeruli (30), proliferative glomerulonephritis (32), tubular necrosis (30, 33, 35, 40, 51, 52), inter-tubular bleeds (5), vascular inflammation (5), tubular hyaline casts formation (5, 35, 37, 40) and dilation of renal tubules (5, 37). Gm nephrotoxicity has been associated with ultrastructural changes in basal membrane of glomeruli and renal tubules as well (53). Gm is filtrated through glomeruli, and during this process, binds to negatively charged structures triggering membrane permeability dysfunction and finally necrosis of tubular cells (54). The essential role of tubular necrosis in Gm-induced renal toxicity is supported by the reversal of renal function after inhibition of tubular necrosis by pre-administration with dipyridamole (33). In addition, dapagliflozin restored structural and functional capacities of kidneys in the rats treated with Gm through inhibiting renal tubular cells apoptosis (5). Vitamin E (α-tocopherol) also corrected functional deficiencies of Gm-exposed kidneys in part through reversing structural changes in glomerular basal membrane and tubules (53). Accordingly, as the role of inflammatory cells in histopathological events in Gm-induced renal injury has been indicated, the administration of anti-inflammatory agents such as *Descurainia sophia* can be beneficial. 

Gm-induced nephrotoxicity is mediated through a complex multifactorial phenomenon with various pathways participating in this process. Among the most important toxic effects of Gm is inducing oxidative stress by overproducing oxidative agents such as reactive oxygen species (ROS) and depressing anti-oxidative capacities in renal cells. Multiple reports have verified the role of oxidative stress in adverse renal effects of Gm in rats (5, 8, 34, 50, 53). Anti-oxidant enzymes and mediators including super oxide dismutase (SOD), glutathione peroxidase, heme oxygenase -1, catalase and glutathione have been depressed in kidneys of Gm-treated rats (6, 29, 30, 32). The rats treated with Gm have shown higher levels of malondialdehyde (MDA), a marker of lipid peroxidation, in renal tissue (29, 32, 50). In fact many protective agents have exerted their effects at least in part by reducing oxidative markers and augmenting anti-oxidative mechanisms (6, 8, 29, 34, 48, 51, 53). In line, the anti-oxidative effects of *Descurainia sophia* have been established in multiple reports (16-18). Propagation of oxidants within renal tissue leads to structural changes in proteins, lipids, DNA, as well as functional exhaustion of critical organelles such as mitochondria (53). Subsequently, these molecular events can trigger adverse pathological outcomes and tubular necrosis as one of the main underlying causes of Gm-induced nephrotoxicity (55). Therefore, using anti-oxidative agents such as *Descurainia sophia* can be helpful to prevent adverse effects of Gm on kidneys. 

Gm also recruits inflammatory mediators to promote its nephrotoxic effects. This is supported by studies that showed beneficial effects of anti-inflammatory agents in preventing Gm-induced AKI (48, 51). Elevated levels of inflammatory cytokines such as interleukin-6 (IL-6), IL-10, and tumor necrosis factor-α (TNF-α) have been described in renal tissue of models of Gm-induced AKI (8, 29, 33). On the other hand, *Descurainia sophia* seeds extract decreased the expression of IL-4, a major marker of type 2 helper T response and inflammation, in mouse model of asthma (21). In mouse model of asthma, *Descurainia sophia* seeds extract also downregulated VEGFa (21). Other protective mechanisms by *Descurainia sophia* may be exerted by encountering the processes initiated by Gm. For example, the plant extract through its active ingredients can modulate signaling pathways such as endoplasmic reticulum stress (i.e. unfolded protein response) (49) and PI3k/Akt/mTOR pathways (22). Helveticoside; an active ingredient of *Descurainia sophia* was shown to role as a potent regulator of intracellular signaling pathways and gene expression (56). Finally, ethanolic extract of *Descurainia sophia* seeds suppressed the activity of cytochrome P450 isoforms (i.e. CYP1A2, CYP2C9, and CYP2C19) which are essential enzymes participating in metabolization of drugs (57-59). Other underlying mechanisms by which *Descurainia sophia* extract and its ingredient suppress adverse effects of Gm on renal tissue are yet to be identified. 

## Conclusion

We showed that *Descurainia sophia* was effective in preventing adverse effects of Gm on renal tissue. This was reflected in reduced serum BUN and creatinine; two indicators of renal toxicity in Wistar rats exposed to Gm for 7 consecutive days. Other biochemical and histological markers indicating protective role of *Descurainia sophia* included reduction in Na+ urinary concentration and FeNa^+^, as well as lower recruitment of inflammatory cells and apoptosis in renal tissue. These effects were dose dependent and were more pronounced in higher dose of *Descurainia sophia* extract. Regarding these observations, and excellent tolerability and safety of this plant, it is applicable to use this plant as a preventive agent in the patients receiving Gm.
